# C3AR1 mRNA as a Potential Therapeutic Target Associates With Clinical Outcomes and Tumor Microenvironment in Osteosarcoma

**DOI:** 10.3389/fmed.2021.642615

**Published:** 2021-03-05

**Authors:** Tiannan Zou, Weibing Liu, Zeyu Wang, Jiayu Chen, Sheng Lu, Kun Huang, Weichao Li

**Affiliations:** ^1^Department of Orthopedic Surgery, The First People's Hospital of Yunnan Province, Affiliated Hospital of Kunming University of Science and Technology, Kunming, China; ^2^Faculty of Medical Science, Kunming University of Science and Technology, Kunming, China; ^3^Yunnan Key Laboratory of Digital Orthopaedics, Kunming, China

**Keywords:** osteosarcoma, C3AR1, mRNA, therapeutic target, prognosis, tumor immune microenvironment

## Abstract

**Objective:** Targeting cancer-specific messenger RNAs (mRNAs) may offer novel insights into therapeutic strategies in osteosarcoma. This study aimed to discover possible osteosarcoma-specific mRNA and probe its biological functions.

**Methods:** Based on mRNA-seq data from the TARGET database, stromal and immune scores were estimated for each osteosarcoma sample via the ESTIMATE algorithm. Stromal and immune mRNAs were obtained via integration of differentially expressed mRNAs between high and low stromal / immune score groups. Among hub and prognostic mRNAs, C3AR1 mRNA was focused and its prognostic value was assessed. The associations between C3AR1 mRNA and immune cells were analyzed via the CIBERSORT algorithm. Its expression was verified in osteosarcoma tissues and cells by RT-qPCR and western blot. The functions of C3AR1 were investigated by a series of experiments.

**Results:** Low stromal and immune scores were both indicative of unfavorable outcomes for osteosarcoma patients. Eighty-eight up-regulated and seven down-regulated stromal and immune mRNAs were identified. Among 30 hub mRNAs, low expression of C3AR1 mRNA indicated worse outcomes than its high expression. There was a lower mRNA expression of C3AR1 in metastatic than non-metastatic osteosarcoma. C3AR1 mRNA was closely correlated to various immune cells such as macrophages. C3AR1 was verified to be down-regulated in osteosarcoma tissues and cells. Its overexpression suppressed proliferation, migration and invasion and induced apoptosis in osteosarcoma cells.

**Conclusion:** C3AR1 mRNA could be a promising therapeutic target for osteosarcoma, linked with prognosis and tumor microenvironment.

## Introduction

Osteosarcoma, as a common malignant bone cancer, is prevailing in childhood and adolescence (1). It has a characteristic of immortal cell proliferation as well as high levels of mRNA translation (2). The main treatment approaches are composed of neoadjuvant chemotherapy, surgery as well as adjuvant chemotherapy (3). It remains unaltered for therapy and prognosis of osteosarcoma in the past 30 years. The 5-year survival rate is over 60% among all cases (4). Nevertheless, for advanced osteosarcoma, the effects of emerging therapies like targeted therapy and immunotherapy are unfavorable (4). Thus, it is of significance for exploiting novel therapies or available signatures to prolong the survival time of osteosarcoma patients.

Growing evidence has emphasized that mRNAs are feasible therapeutic targets in cancer therapy (5). Alterations in abundant cancer-specific mRNAs may activate oncogenes and inactivate tumor-suppressor genes, thereby facilitating tumor progression (5). The concept about RNA-targeted therapy was put forward in 1978. Since there are no risks of insertion mutagenesis, it is attractive to use mRNAs in place of DNAs as therapeutic substances (6). In comparison to protein or peptide delivery, mRNAs can extend the practicality of effective molecules (7). It is challenging to develop the effective therapy without adverse effects in cancers. Hence, it is of significance to understand the functional implications of cancer-specific mRNAs in osteosarcoma biology.

Tumor microenvironment is composed of immune and stromal cells. Stromal and immune mRNAs are critical factors for prognosis of osteosarcoma (4). Therefore, the comprehensive analysis of the correlation between immune-related gene signatures and overall survival may shed light on pathogenesis of osteosarcoma. Manipulating the tumor immune microenvironment may be a key part of various therapeutic applications for cancers (8). Nucleic acid-based approaches particularly mRNA is required and exploited for immunotherapies. *In vivo* research has offered attractive demonstrations concerning the feasibility of mRNA-based immunotherapy (9, 10). Herein, we identified a novel stromal and immune prognostic mRNA, which could be a promising therapeutic target in osteosarcoma.

## Materials and Methods

### Data Acquisition and Preprocessing

mRNA-seq data and matched clinical information of osteosarcoma were downloaded from the TARGET database (https://ocg.cancer.gov/programs/target) on November 3, 2020. Genes that encode proteins were annotated using Homo sapiens gene annotation files from the Ensemble database (http://www.ensembl.org/index.html). Following removing samples without complete clinical information. Eighty-six osteosarcoma samples were obtained for this study. Sample information was merged with mRNA-seq.

### Estimation of STromal and Immune Cells in MAlignant Tumor Tissues Using Expression Data

Stromal and immune levels were estimated for each osteosarcoma sample based on the mRNA-seq data using the ESTIMATE software (11). The stromal and immune cell scores were then calculated for each sample. All osteosarcoma samples were, respectively, separated into high and low stromal/immune cell score groups according to the median values. Kaplan-Meier curves were conducted for the survival time between groups and differences in survival probability were assessed via log-rank test using the Survminer package in R. The associations between stromal or immune cell scores and other clinicopathological characteristics including age, diagnosis, gender, race, primary tumor site and specific tumor site were estimated by Wilcoxon rank-sum test or Kruskal–Wallis test.

### Differential Expression Analyses

To screen mRNAs related to stromal and immune scores, differential expression analyses were presented between high and low stromal or immune score groups. The criteria for differentially expressed mRNAs were as follows: |log 2 fold change (FC)| > 1 and false discovery rate (FDR) < 0.01. These mRNAs were visualized into heatmaps. Then, up- and down-regulated stromal and immune mRNAs were separately obtained.

### Functional Annotation Analyses

Gene Ontology (GO) and Kyoto Encyclopedia of Genes and Genomes (KEGG) functional annotation analyses were presented based on differentially expressed stromal and immune mRNAs via the Database for Annotation, Visualization, and Integrated Discovery (DAVID; http://www.david.niaid.nih.gov) (12). GO terms were composed of biological process (BP), cellular component (CC), as well as molecular function (MF).

### Protein-Protein Interaction

Differentially expressed stromal and immune mRNAs were imported into the Search Tool for the Retrieval of Interacting Genes Database (STRING) database (http://string-db.org/) (13). A PPI network was visualized by the Cytoscape software (https://cytoscape.org/) (14). Hub mRNAs were identified according to degrees.

### Univariate Cox Regression Analysis

Univariate cox regression analysis was presented to analyze the associations between differentially expressed stromal and immune mRNAs and prognosis among osteosarcoma.

### CIBERSORT

The infiltration levels of 22 immune cells in osteosarcoma samples were estimated by the CIBERSORT algorithm (http://cibersort.stanford.edu/) (15). The differences in the infiltration levels of these immune cells were compared between osteosarcoma and normal samples via the Wilcoxon rank-sum test.

### Osteosarcoma Tissues

Ten paired osteosarcoma and normal tissue specimens were collected from the Department of Orthopedic Surgery of The First People's Hospital of Yunnan Province (Kunming, China). This research followed the Declaration of Helsinki and gained the approval of the Ethics Committee of The First People's Hospital of Yunnan Province (2020048). All subjects provided written informed consent.

### RT-qPCR

RNA extraction was achieved through TRIZOL reagent (Beyotime, Beijing, China). RNA was reverse transcribed into cDNA. qPCR was presented by SYBR Green Master kit (Roche, Switzerland). The primer sequences were as follows: C3AR1, 5′-CCCTACGGCAGGTTCCTATG-3′ (forward) and 5′-GACAGCGATCCAGGCTAATGG-3′ (reverse), GAPDH, 5′-ACAACTTTGGTATCGTGGAAGG-3′ (forward) and 5′-GCCATCACGCCACAGTTTC-3′ (reverse). C3AR1 expression was quantified with the 2^−ΔΔCt^ method.

### Cell Culture and Transfection

Human osteosarcoma cell lines Saos-2 and U-2OS (ATCC, USA) and osteoblast hFOB 1.19 were cultured in RPMI-1640 medium plus 10% fetal bovine serum (FBS; Beyotime). Plasmid (Genepharma, Shanghai, China) and controls were utilized for overexpressing C3AR1. Saos-2 and U-2OS cells were transfected with 100 nM RNA oligonucleotides via Lipofectamine 2000 (Beyotime).

### Western Blot

Total protein was extracted via RIPA buffer, which was evaluated by BCA kit (Beyotime). Samples were separated by SDS-PAGE and transferred onto PVDF membrane. Following being blocked, membrane was incubated by anti-C3AR1 (1:1,000; ab126250; Abcam, USA) or anti-GAPDH (1:1,000; ab8245) at 4°C overnight, followed by being probed with secondary antibody (1:5,000; ab7090). The protein bands were then visualized through ECL Plus substrate (Beyotime).

### Clone Formation Assay

Transfected cells were grown in a 6-well plate (1 × 10^3^/well) for 2 weeks. Following being washed by PBS, cells were fixed by paraformaldehyde and stained by 0.5% crystal violet. The images were observed under a microscope (Olympus, Japan).

### Flow Cytometry

Cells were centrifuged at 2,000 g at 4°C. Then, supernatant samples were removed. Cells were suspended by binding buffer and incubated by Annexin V-FITC and propidium iodide. The apoptotic level was assessed via Annexin-V-FITC detection kits (Solarbio, Beijing, China) on flow cytometry.

### Scratch Assay

Transfected cells were planted into a 6-well plate for 24 h. A mark perpendicular to the bottom was drawn. Cells were incubated by serum-free medium. After 0, 24, and 48 h, images were investigated under a microscope (Olympus, Japan).

### Transwell Assay for Invasion

Transfected cells were seeded onto the upper chamber. Matrigel was added to transwell inserts lasting 2 h. FBS were added to the lower chamber. After 24 h, non-invasive cells were removed through a cotton swab. Invasive cells were stained by crystal violet.

### Statistical Analysis

All analysis was conducted by R language or GraphPad Prism software. The correlation between the levels of immune cells was analyzed by Spearson correlation analysis. Moreover, Spearson correlation analysis was presented whether C3AR1 mRNA expression exhibited associations with the levels of immune cells. |*r*| ≥ 0.8 suggests an extremely strong correlation between the two variables; 0.6 ≤ |*r*| < 0.8 indicates strong correlation; 0.4 ≤ |*r*| < 0.6 indicates moderate correlation; 0.2 ≤ |*r*| < 0.4 represents a weak correlation; |*r*| < 0.2 shows extremely weak. For experiments, data were expressed as mean ± standard deviation. Comparisons between groups were analyzed by student's *t*-test or one-way analysis of variance. *P* < 0.05 was statistically significant.

## Results

### Stromal and Immune Scores Closely Link With Clinical Outcomes and Clinicopathological Characteristics in Osteosarcoma

**Figure 1:**
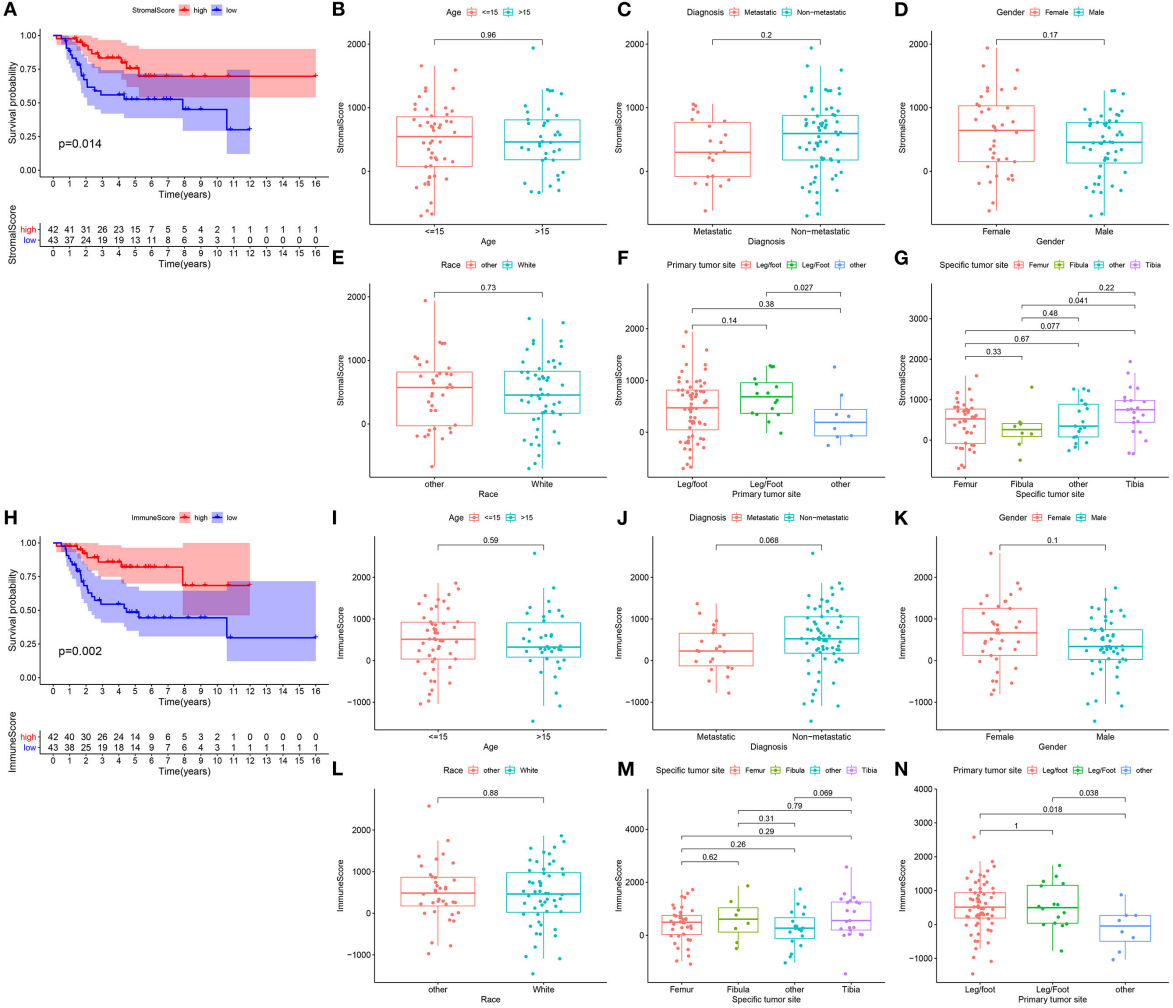
Stromal and immune scores are correlated to prognosis and clinicopathological characteristics in osteosarcoma. **(A)** Kaplan-Meier of overall survival probability for high (red) and low (blue) stromal score osteosarcoma subgroups. **(B–G)** Box plots for the association between stromal score and clinicopathological characteristics including age **(B)**, metastasis **(C)**, gender **(D)**, race **(E)**, primary tumor site **(F)** and specific tumor site **(G)**. **(H)** Kaplan-Meier of overall survival probability for high (red) and low (blue) immune score groups. **(I–N)** Box plots for the association between immune score and clinicopathological characteristics including age **(I)**, metastasis **(J)**, gender **(K)**, race **(L)**, specific tumor site **(M)** and primary tumor site **(N)**.

In this study, we estimated the stromal and immune scores for each osteosarcoma sample from the TARGET database via the ESTIMATE algorithm. As depicted in Kaplan-Meier curves, patients with low stromal scores experienced poorer survival time than those with high scores (*p* = 0.014; Figure 1A). The association between stromal scores and clinicopathological characteristics was probed in depth. No significant differences between stromal scores and age (*p* = 0.96; Figure 1B), metastasis (*p* = 0.2; Figure 1C), gender (*p* = 0.17; Figure 1D) and race (*p* = 0.73; Figure 1E) were detected between high and low stromal score patients. There was a distinct difference in stromal levels among primary tumor sites (Figure 1F). For specific tumor sites, stromal scores were markedly elevated in tibia than fibular (*p* = 0.041; Figure 1G). Meanwhile, we analyzed the clinical implications of immune scores in osteosarcoma. In Figure 1H, high immune scores indicated prolonged survival time than low immune scores (*p* = 0.002). There was no significance in age (*p* = 0.59; Figure 1I), metastasis (*p* = 0.068; Figure 1J), gender (*p* = 0.1; Figure 1K), race (*p* = 0.88; Figure 1L) and specific tumor sites (Figure 1M) between high and low immune score groups. Among different primary tumor sites, immune scores were statistically significant (Figure 1N). Above findings revealed the clinical implications of stromal and immune scores in osteosarcoma.

### Differentially Expressed Stromal and Immune mRNAs in Osteosarcoma

**Figure 2:**
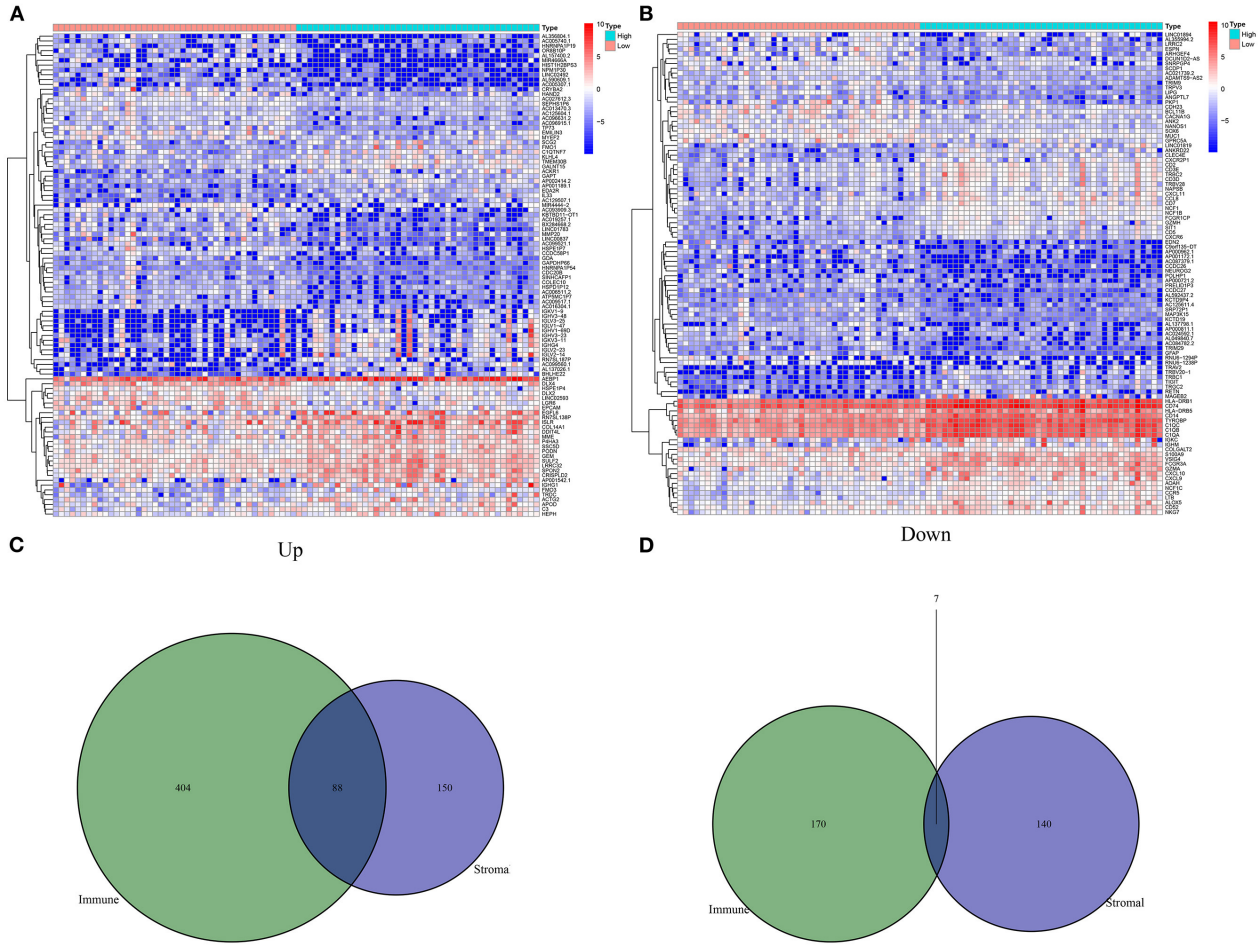
Screening differentially expressed stromal and immune mRNAs in osteosarcoma. **(A)** Heat map for differentially expressed mRNAs between high and low stromal score groups. **(B)** Heat map for differentially expressed mRNAs between high and low immune score groups. **(C)** Venn diagram for up-regulated stromal and immune mRNAs. **(D)** Venn diagram for down-regulated stromal and immune mRNAs.

With the cutoff of |log 2 FC| > 1 and FDR < 0.01, 385 differentially expressed stromal mRNAs were screened between high and low stromal score groups (Figure 2A). Meanwhile, 669 immune mRNAs were dysregulated between high and low immune score groups (Figure 2B). Because both stromal and immune score exhibited important clinical implications, we focused on the analysis of stromal and immune mRNAs. Following overlapping above two lists of mRNAs, we obtained 88 up-regulated stromal and immune mRNAs (Figure 2C). Additionally, 7 down-regulated stromal and immune mRNAs were acquired in osteosarcoma (Figure 2D). Supplementary Table 2 listed all abnormally expressed stromal and immune mRNAs. These mRNAs could be linked with stromal and immune levels in osteosarcoma.

### Biological Functions of Stromal and Immune mRNAs

**Figure 3:**
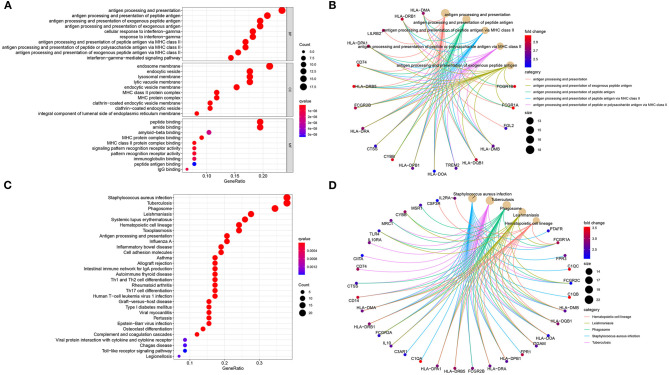
Biological functions of stromal and immune mRNAs. **(A)** The top ten biological processes (BP) cellular components (CC) and molecular functions (MF), respectively. **(B)** The top five biological processes and enriched mRNAs. **(C)** Bubble chart for the top 30 signaling pathways. **(D)** The top five KEGG pathways and enriched mRNAs.

The functional functions of stromal and immune mRNAs were probed in depth. GO enrichment analysis suggested that these mRNAs were distinctly enriched in antigen processing and presentation-related processes (Figure 3A). Furthermore, they were involved in MHC protein complex formation and had the MHC protein complex binding molecular function. Figure 3B showed the most enriched biological processes and corresponding mRNAs. As depicted in the KEGG enrichment analysis, immune-related pathways were distinctly enriched such as hematopoietic cell lineage, phagosome, antigen processing and presentation, Th1, Th2, and Th17 cell differentiations (Figures 3C,D). Hence, these mRNAs could participate in modulating immune pathways in osteosarcoma.

### Hub and Prognostic mRNAs for Osteosarcoma

**Figure 4:**
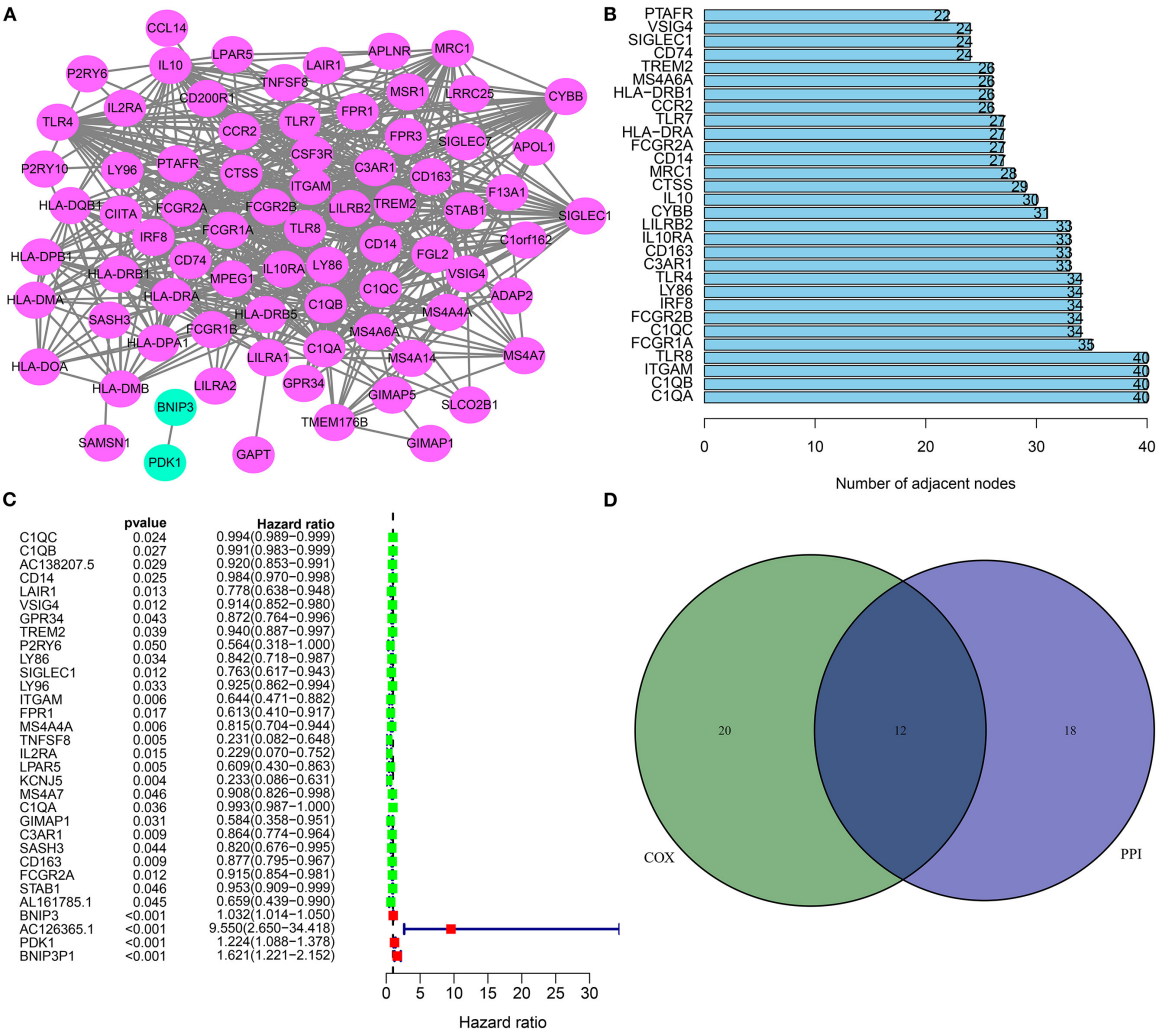
Hub and prognosis-related mRNAs for osteosarcoma. **(A)** A PPI network based on differentially expressed stromal and immune mRNAs. **(B)** The 30 hub genes according to degrees. **(C)** Forest diagram for 32 prognosis-related stromal and immune mRNAs according to univariate cox regression analysis. **(D)** Venn diagram for 12 hub and prognosis-related stromal and immune mRNAs.

Based on these stromal and immune mRNAs, a PPI network was established, as shown in Figure 4A. There were 77 nodes in the network. Among them, 76 mRNAs were up-regulated in high stromal and immune score group. Figure 4B listed the top 30 mRNAs according to degrees, which were considered as hub mRNAs. Univariate cox regression analysis showed that 32 stromal and immune mRNAs had significant correlations to prognosis of osteosarcoma (Figure 4C). After integration of hub mRNAs and prognosis-related mRNAs, we obtained 12 hub and prognosis-related mRNAs (C1QA, C1QB, ITGAM, C1QC, LY86, C3AR1, CD163, CD14, FCGR2A, TREM2, SIGLEC1, and VSIG4) for osteosarcoma (Figure 4D), which could play key roles in osteosarcoma progression.

### Low C3AR1 mRNA Expression Is Correlated to Poor Prognosis and Metastasis in Osteosarcoma

**Figure 5:**
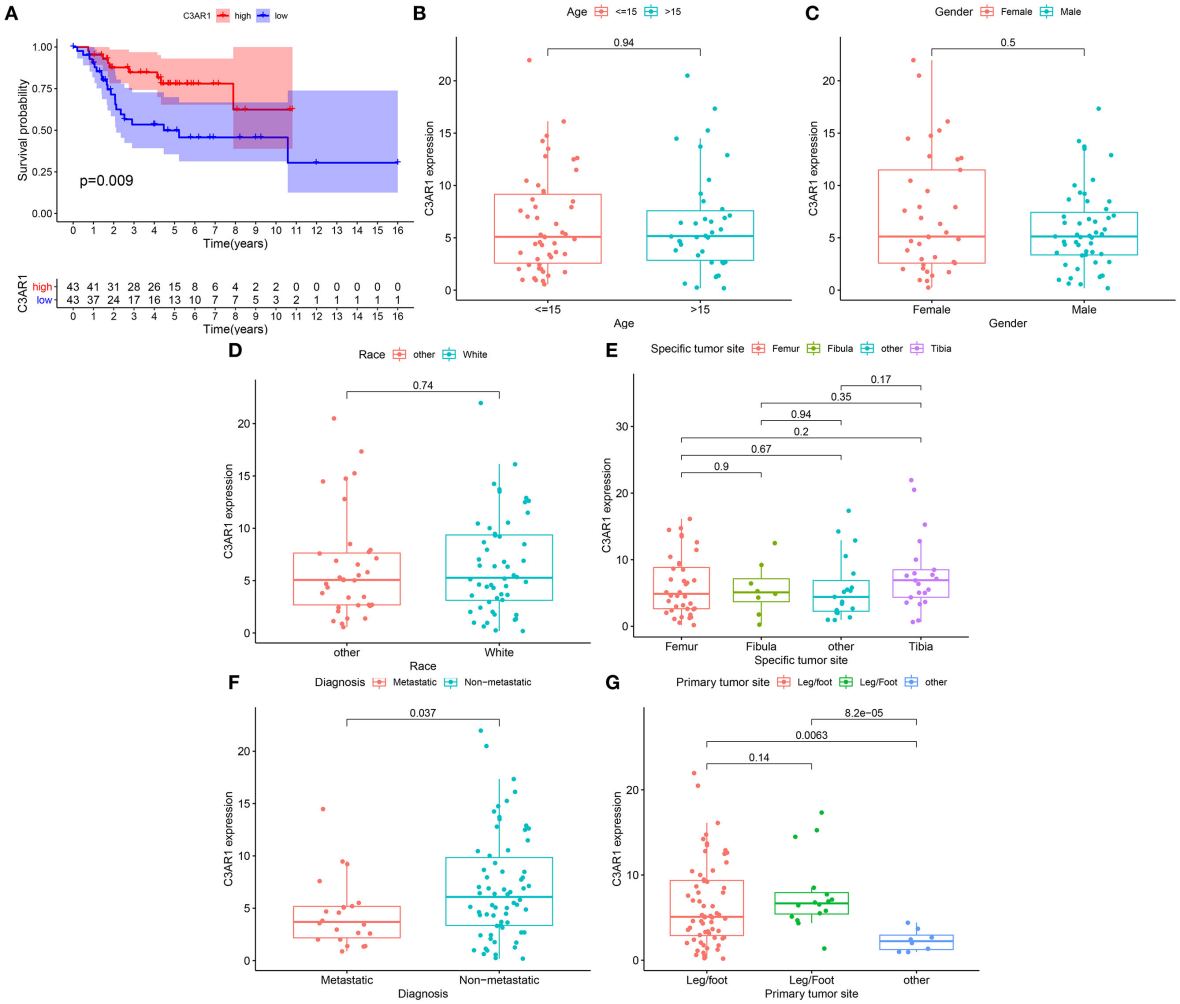
Association between C3AR1 mRNA expression and prognosis and clinicopathological characteristics among osteosarcoma. **(A)** Kaplan-Meier of overall survival between high (red) and low (blue) C3AR1 mRNA expression osteosarcoma groups. **(B–G)** The association between C3AR1 mRNA expression and age **(B)**, gender **(C)**, race **(D)**, specific tumor site **(E)**, metastasis **(F)** and primary tumor sites **(G)**.

Our Kaplan-Meier curves showed that patients with high C3AR1 expression had prolonged survival time in comparison to those with its low expression (*p* = 0.009; Figure 5A). We analyzed the correlation between C3AR1 expression and clinicopathological characteristics among osteosarcoma patients. There was no significant correlation between C3AR1 expression and age (Figure 5B), gender (Figure 5C), race (Figure 5D), and specific tumor site (Figure 5E). C3AR1 expression was distinctly decreased in metastatic compared to non-metastatic osteosarcoma patients (*p* = 0.037; Figure 5F). Compared to others, there was a distinct difference in C3AR1 expression between primary tumor sites (Figure 5G). Thus, lowly expressed C3AR1 mRNA could be correlated to poor prognosis and metastasis in osteosarcoma.

### Landscape of Immune Cell Components in Osteosarcoma

**Figure 6:**
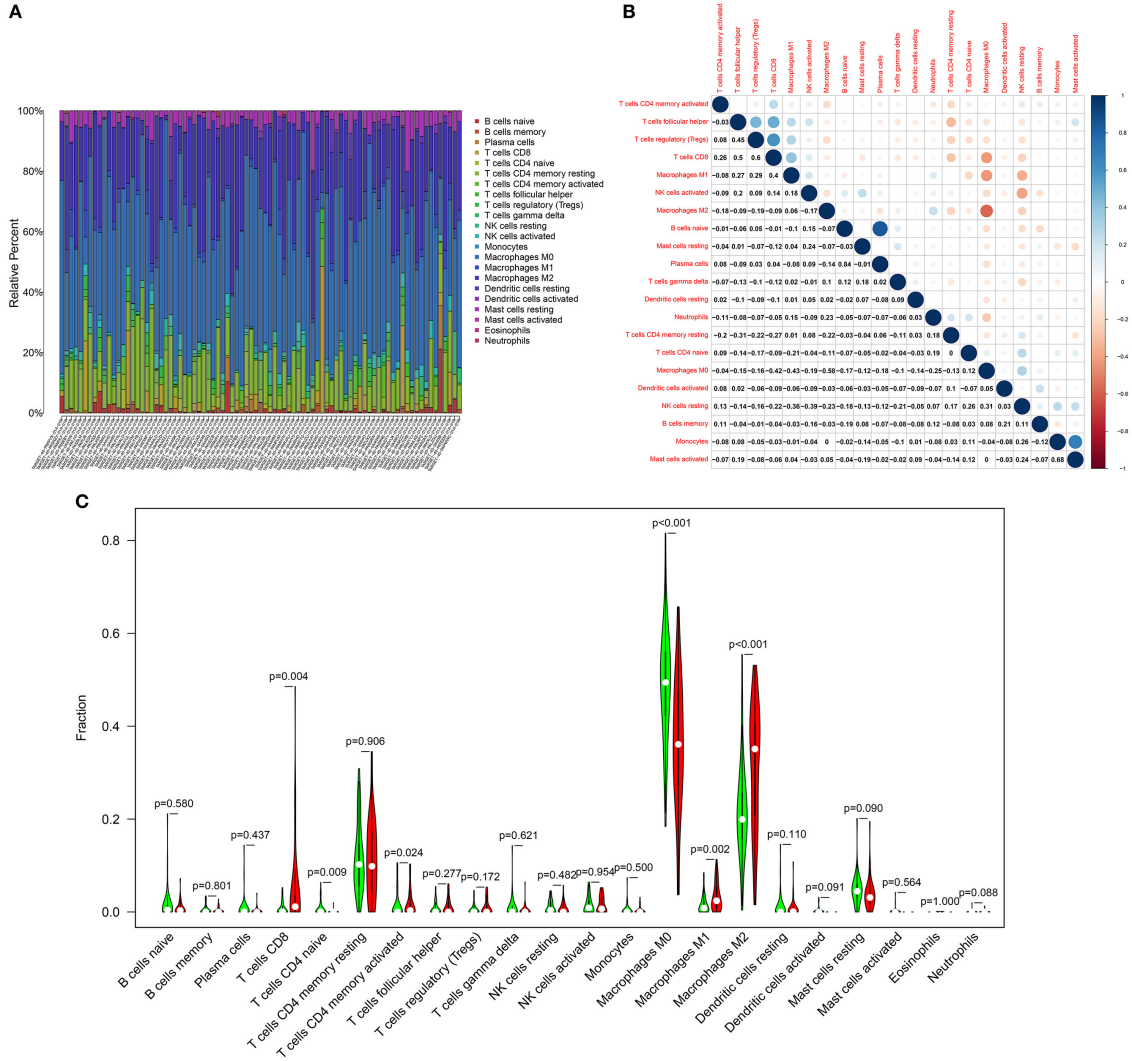
Landscape of immune cell components in osteosarcoma. **(A)** Stacked graph for the proportion of various types of immune cells in osteosarcoma tissues. **(B)** Heat map for the correlations between the levels of immune cells among osteosarcoma samples. **(C)** Violin diagram for the levels of immune cells between osteosarcoma (red) and normal (green) samples.

The CIBERSORT algorithm was utilized for examining the relative proportions of immune cells in each osteosarcoma specimen. Figure 6A showed the heterogeneity in levels of B cells naïve, B cells memory, plasma cells, T cells CD8, T cells CD4 naïve, T cells CD4 memory resting, T cells CD4 memory activated, T cells follicular helper, T cells regulatory (Tregs), T cells gamma delta, NK cells resting, NK cells activated, monocytes, macrophages M0, macrophages M1, macrophages M2, dendritic cells resting, dendritic cells activated, mast cells resting, mast cells activated, eosinophils and neutrophils among osteosarcoma tissues. Positive and negative correlations between immune cells were observed, as shown in Figure 6B. There was a strong correlation between mast cells activated and monocytes (*r* = 0.68) in osteosarcoma. Plasma cells were extremely strongly correlated with B cells naïve (*r* = 0.84). Macrophages M0 had moderate and negative links with macrophages M2 (*r* = −0.58), macrophages M1 (*r* = −0.43), T cells CD8 (*r* = −0.43). T cells CD8 were positively and moderately linked with Tregs (*r* = 0.6) and T cells follicular helper (*r* = 0.5). Above correlation analysis between different kinds of immune cells revealed that there was the crosstalk between immune cells. We further analyzed the differences in immune cell levels between osteosarcoma and normal tissues. The data showed that the levels of T cells CD8 (*p* = 0.004), T cells CD4 memory activated (*p* = 0.024), macrophages M1 (*p* = 0.002) and macrophages M2 (*p* < 0.001) were markedly higher in osteosarcoma than normal tissues (Figure 6C). In contrast, T cells CD4 naïve (*p* = 0.009) and macrophages M0 (*p* < 0.001) had lowered levels in osteosarcoma compared to normal tissues.

### C3AR1 mRNA Is Moderately Correlated With Tumor Immune Microenvironment in Osteosarcoma

**Figure 7:**
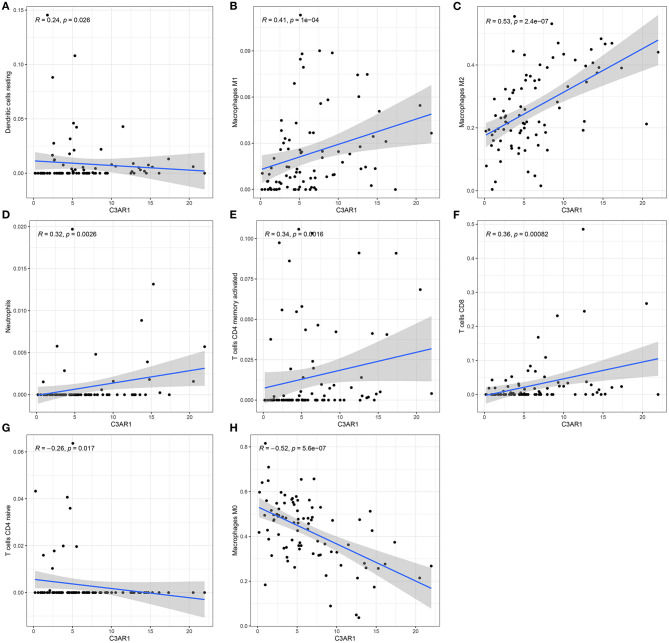
C3AR1 mRNA expression associates with distinct kinds of immune cells in osteosarcoma. There were significant correlations between C3AR1 expression and **(A)** dendritic cells, **(B)** macrophages M1, **(C)** macrophages M2, **(D)** neutrophils, **(E)** T cells CD4 memory activated, **(F)** T cells CD8, **(G)** T cells CD4 naïve and **(H)** macrophages M0.

The association between C3AR1 mRNA expression and immune cells was further analyzed. As a result, C3AR1 mRNA expression had a weak correlation with dendritic cells resting levels (*r* = 0.24, *p* = 0.026; Figure 7A). C3AR1 expression was moderately correlated to macrophages M1 (*r* = 0.41, *p* = 1e-04; Figure 7B) and macrophages M2 (*r* = 0.53, *p* = 2.4e-07; Figure 7C). Meanwhile, there were weak associations between C3AR1 expression and neutrophils (*r* = 0.32; *p* = 0.0026; Figure 7D), T cells CD4 memory activated (*r* = 0.34; *p* = 0.0016; Figure 7E), and T cells CD8 (*r* = 0.36; *p* = 0.00082; Figure 7F). A weak negative correlation between C3AR1 expression and T cells CD4 naïve levels was found among osteosarcoma samples (*r* = −0.26; *p* = 0.0017; Figure 7G). In Figure 7H, C3AR1 expression was moderately and negatively associated with macrophages M0 (*r* = −0.52; *p* = 5.6e-07). Collectively, C3AR1 expression may associate with tumor immune microenvironment in osteosarcoma.

### Down-Regulation of C3AR1 in Osteosarcoma

**Figure 8:**
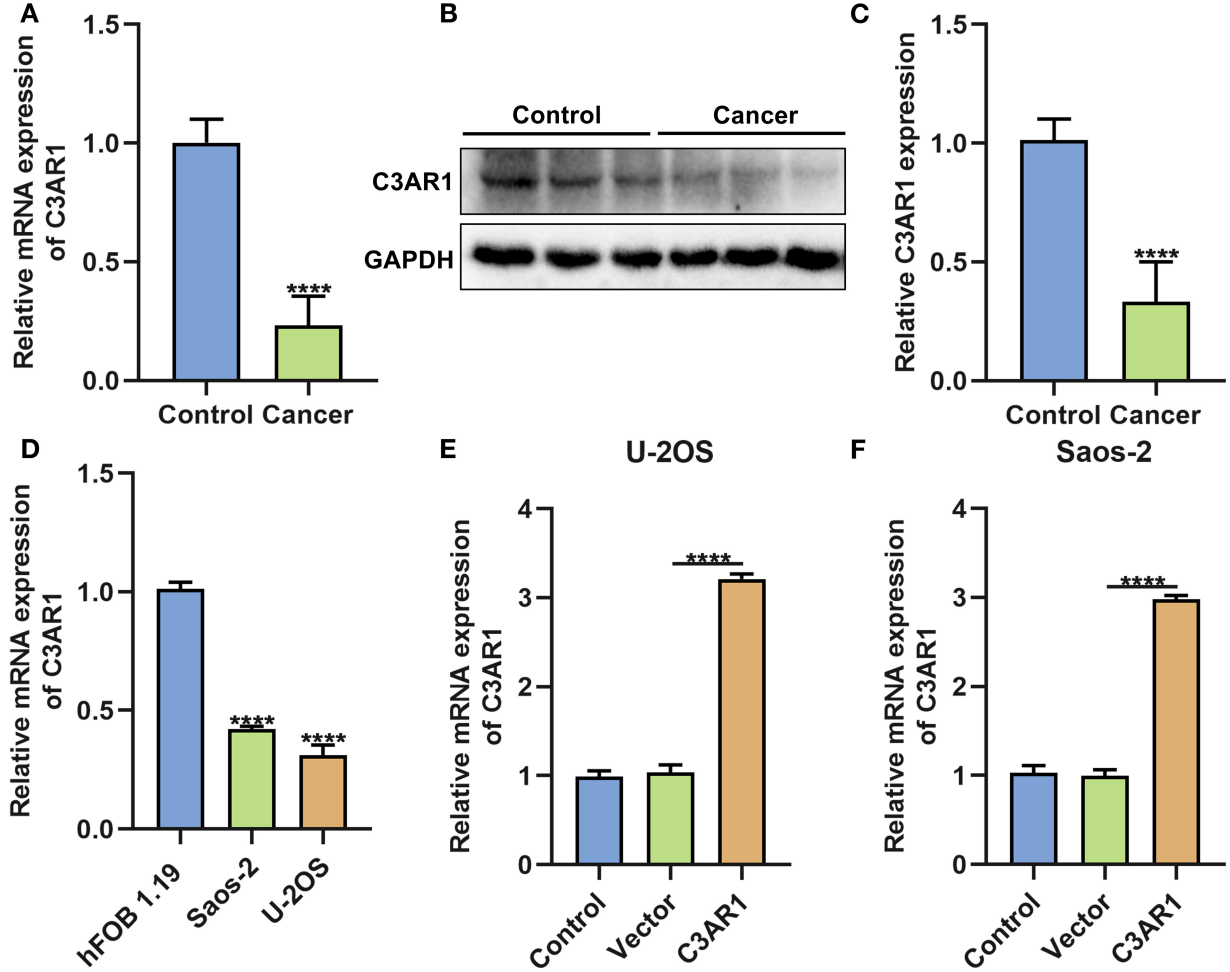
C3AR1 down-regulation in osteosarcoma. **(A)** RT-qPCR and **(B,C)** western blot for C3AR1 expression in osteosarcoma and control tissue specimens. **(D)** RT-qPCR for C3AR1 expression in osteosarcoma cell lines Saos-2 and U-2OS and osteoblast hFOB 1.19. **(E,F)** RT-qPCR for C3AR1 expression in Saos-2 and U-2OS cells after overexpressing C3AR1. ****p < 0.0001.

C3AR1 was further verified in osteosarcoma tissues and cells. Our data confirmed that C3AR1 mRNA (Figure 8A) and protein (Figures 8B,C) were both lowered in cancer than normal tissue specimens (both *p* < 0.0001). Also, its down-regulation was detected in osteosarcoma cells (Saos-2 and U-2OS) compared to normal osteoblast hFOB 1.19 cells (Figure 8D; both *p* < 0.0001). To investigate the biological functions of C3AR1, it was successfully overexpressed in Saos-2 and U-2OS cells (Figures 8E,F; both *p* < 0.0001).

### C3AR1 Overexpression Suppresses Proliferation and Induces Apoptosis in Osteosarcoma Cells

**Figure 9:**
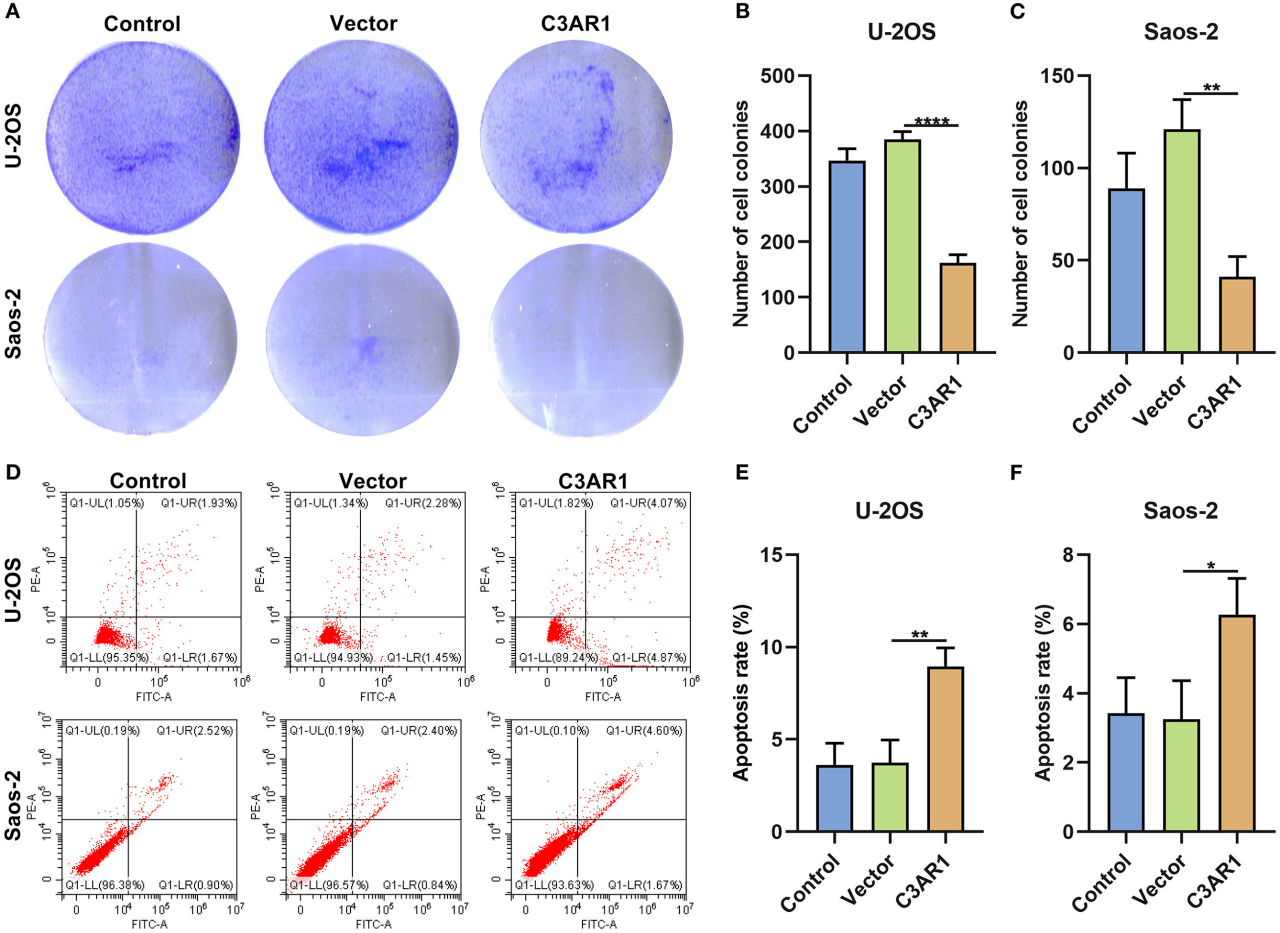
C3AR1 overexpression suppresses proliferation and induces apoptosis in osteosarcoma cells. **(A–C)** Number of cell colonies of Saos-2 and U-2OS cells with C3AR1 overexpression. **(D–F)** Flow cytometry for apoptotic levels in Saos-2 and U-2OS cells with C3AR1 overexpression. *p < 0.05; **p < 0.01; ****p < 0.0001.

After overexpressing C3AR1, proliferation and apoptosis of osteosarcoma cells were evaluated. We found that C3AR1 overexpression distinctly reduced number of colonies of Saos-2 (*p* < 0.0001) and U-2OS cells (*p* < 0.01; Figures 9A–C). Moreover, apoptotic levels were markedly lessened following overexpressing C3AR1 for Saos-2 (*p* < 0.01) and U-2OS cells (*p* < 0.05; Figures 9D–F).

### C3AR1 Overexpression Inhibits Migration and Invasion of Osteosarcoma Cells

**Figure 10:**
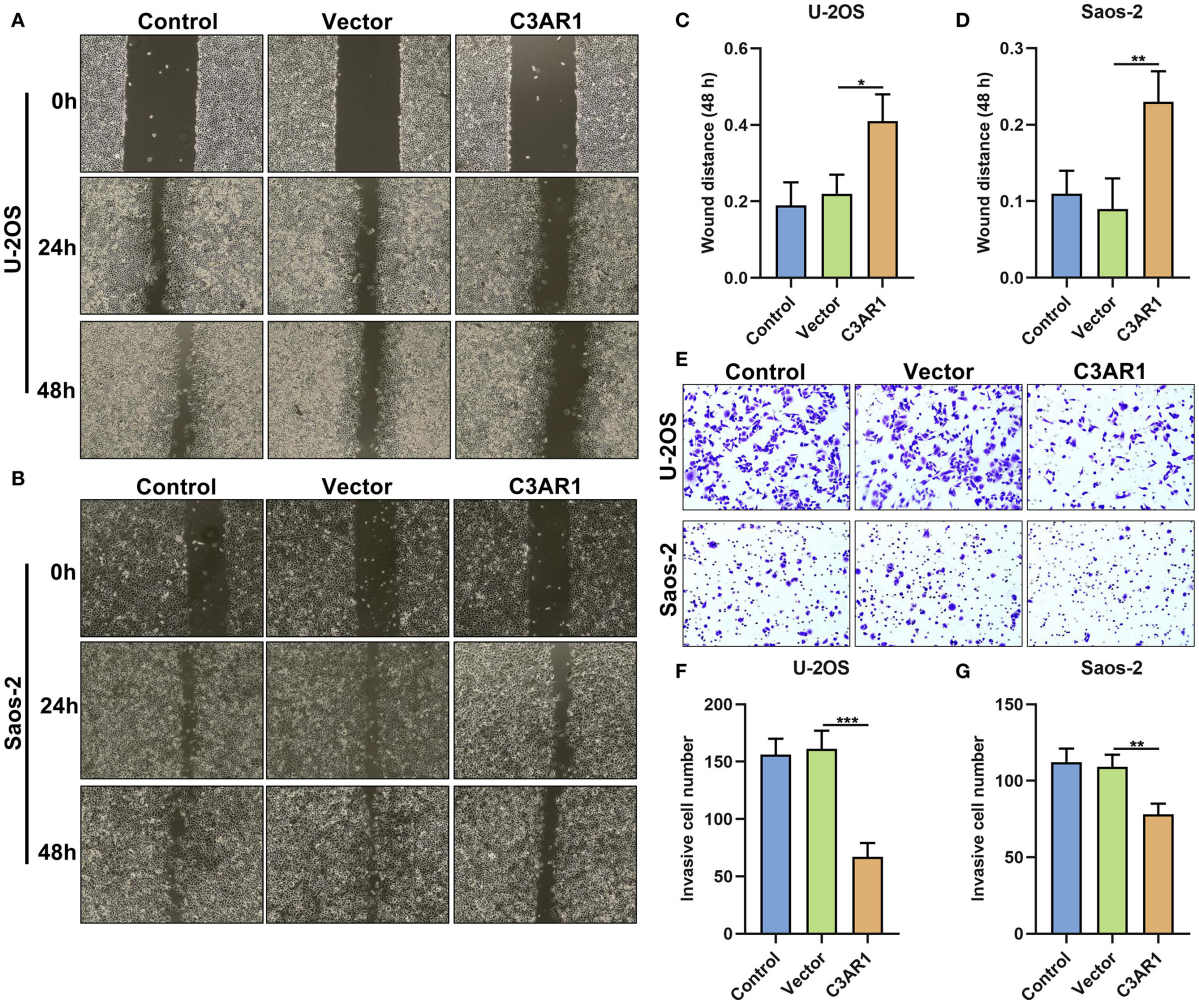
C3AR1 overexpression reduces migration and invasion of osteosarcoma cells. **(A–D)** Assessment of wound distance of Saos-2 and U-2OS cells with C3AR1 overexpression. **(E–G)** Number of invasive Saos-2 and U-2OS cells with C3AR1 overexpression. *p < 0.05; **p < 0.01; ***p < 0.001.

Migrated and invasive capacities of osteosarcoma cells were observed after overexpressing C3AR1. This study showed that wound distance was markedly wider in Saos-2 (*p* < 0.05) and U-2OS cells (*p* < 0.01) transfected with C3AR1 overexpression (Figures 10A–D). Furthermore, C3AR1 overexpression decreased the number of invasive Saos-2 (*p* < 0.001) and U-2OS cells (*p* < 0.01; Figures 10E–G).

## Discussion

This study revealed the clinical implications of stromal and immune scores in osteosarcoma. We identified 12 critical stromal and immune mRNAs that exhibited tight correlations with outcomes of osteosarcoma. Among them, C3AR1 mRNA was associated with prognosis, metastasis, and tumor immune microenvironment in osteosarcoma. Hence, C3AR1 mRNA might be a promising therapeutic target for osteosarcoma.

The ESTIMATE algorithm has been used for various cancers, suggesting that it is effective and robust based on the expression profiles. Utilizing this algorithm, Ke et al. identified novel immune-related genes such as LINC01564, LINC02208 and ODAM for testicular cancer (16). Wang et al. developed a stromal and immune score-related gene signature composed of SOX9, LRRC32, CECR1, and MS4A4A for gastric cancer (17). Zhou et al. screened prognostic genes related to the tumor microenvironment in stomach adenocarcinoma (18). Herein, we estimated stromal and immune levels based on the ESTIMATE algorithm. Our results showed that stromal and immune scores were both linked with outcomes for osteosarcoma patients. Consistent with a previous study, high stromal and immune scores were both indicative of prolonged survival time (18). These data demonstrated that tumor microenvironment exhibited a distinct correlation with prognosis of osteosarcoma.

Eighty-eight up- and seven down-regulated stromal and immune mRNAs were identified in osteosarcoma. They were mainly involved in several immune pathways such as antigen processing and presentation, MHC protein complex, Th1, Th2, and Th17 cell differentiation and the like. For instance, antigen presentation may be decreased in osteosarcoma than healthy bones (19). We established a PPI network based on these stromal and immune mRNAs. Thirty hub mRNAs were screened. Among them, 12 mRNAs were distinctly correlated to outcomes of osteosarcoma patients. We found that C3AR1 mRNA could be a predictive factor for prognosis of osteosarcoma patients. After validation, C3AR1 was down-regulated in osteosarcoma tissues and cells. As previous studies, C3AR1 mRNA overexpression in the early stages of acute myeloid leukemia could predict shorter survival time (20). Furthermore, it is involved in ductal carcinoma *in situ* progression (21). C3AR1 is a hub mRNA for multiple myeloma (22), colon cancer (23) and melanoma (24). C3AR1 may predict chemotherapy resistance and outcomes of soft tissue sarcomas (25). Metastatic osteosarcoma leads to poor clinical outcomes, especially lung metastases. The 5-year survival rates are <30% toward patients with osteosarcoma metastasized to lungs (26). Increasing evidence has highlighted that the biological drivers of metastatic phenotype of osteosarcoma have a distinction from primary tumors (27). Our data suggested that C3AR1 mRNA was closely linked with metastatic progression. Its overexpression suppressed proliferation, migration and invasion and induced apoptosis of osteosarcoma cells. Collectively, C3AR1 mRNA could be a prognostic marker for prognosis and metastasis in osteosarcoma.

The CIBERSORT algorithm was utilized for detection of the relative proportions of immune cells in each osteosarcoma specimen. As a previous study, utilizing the CIBERSORT algorithm, macrophages could be primary infiltrating immune cells in osteosarcoma samples, especially in macrophages M0 and M2 (28). Our data demonstrated that there were positive and negative correlations between immune cells in osteosarcoma. There was a strong correlation between mast cells activated and monocytes, between plasma cells and B cells naïve, between macrophages M0 and macrophages M2/M1, between T cells CD8 and Tregs/T cells follicular helper, indicating that there was the crosstalk between immune cells in tumor microenvironment. Furthermore, we found that the levels of T cells CD8, T cells CD4 memory activated, macrophages M1 and macrophages M2 were markedly higher in osteosarcoma than normal tissues. Oppositely, T cells CD4 naïve and macrophages M0 had lowered levels in osteosarcoma compared to normal tissues. These findings indicated that immune cell infiltrates could be linked with osteosarcoma progression.

Previously, C3AR1 is closely associated with immune responses. For example, C3AR1 is associated with immune infiltration in sepsis (29). Inactivated C3AR1 could reverse an abnormal immune network in Alzheimer's disease (30). Endothelial C3AR1 regulates vascular inflammatory response in aging or neurodegenerative diseases (31). Our results showed that C3AR1 mRNA expression had a weak correlation with dendritic cells resting levels. Moreover, it was moderately correlated to macrophages M1 and macrophages M2. C3AR1 is a major effector for macrophages-regulated fibrosing steatohepatitis (32). Meanwhile, there were weak associations between C3AR1 expression and neutrophils, T cells CD4 memory activated, and T cells CD8. It has been found that C3AR1 may control the mobilization of neutrophils after spinal cord damage (33). Autocrine or paracrine C3AR1 correlates Toll-like receptor 2 stimulation with maturation of dendritic cells, thereby promoting the response of effector T cells (34). Inactivation of C3AR1 suppresses expansion and differentiation for alloreactive T cells CD8 (35). There was a weak negative correlation between C3AR1 expression and T cells CD4 naïve levels in osteosarcoma samples. C3AR1 expression was moderately and negatively associated with macrophages M0 levels. Hence, C3AR1 mRNA may be linked with tumor immune microenvironment in osteosarcoma.

Taken together, our study identified a novel stromal- and immune-related C3AR1 mRNA, which could be tightly correlated to outcomes of osteosarcoma patients. C3AR1 mRNA might be a feasible therapeutic target in osteosarcoma therapy.

## Conclusion

This study showed that stromal and immune scores were distinctly correlated with prognosis of osteosarcoma. We further screened stromal and immune prognostic mRNAs. Among them, C3AR1 mRNA was focused, which was associated with outcomes and metastasis in osteosarcoma. Also, there was a closely correlation between C3AR1 mRNA and immune cells, indicating that C3AR1 mRNA could be involved in regulation of tumor immune microenvironment. Hence, C3AR1 mRNA could be an underlying therapeutic target for osteosarcoma.

## Data Availability Statement

The original contributions presented in the study are included in the article/Supplementary Material, further inquiries can be directed to the corresponding author/s.

## Ethics Statement

The studies involving human participants were reviewed and approved by the Ethics Committee of The First People's Hospital of Yunnan Province (2020048). The patients/participants provided their written informed consent to participate in this study.

## Author Contributions

KH and WL conceived and designed the study. TZ and WL conducted most of the experiments and data analysis, and wrote the manuscript. ZW, JC, and SL participated in collecting data and helped to draft the manuscript. All authors contributed to the article and approved the submitted version.

## Conflict of Interest

The authors declare that the research was conducted in the absence of any commercial or financial relationships that could be construed as a potential conflict of interest.

## Supplementary Material

Supplementary Table 1 Clinical information of 86 osteosarcoma samples from the TARGET database.

Supplementary Table 2 Differentially expressed stromal and immune mRNAs in osteosarcoma.

## Abbreviations

mRNAs: messenger RNAs
ESTIMATE: Estimation of STromal and Immune cells in MAlignant Tumor tissues using Expression data
FC: fold change
FDR: false discovery rate
GO: Gene Ontology
KEGG: Kyoto Encyclopedia of Genes and Genomes
DAVID: Database for Annotation, Visualization, and Integrated Discovery
BP: biological process
CC: cellular component
MF: molecular function
PPI: protein-protein interaction
STRING: Search Tool for the Retrieval of Interacting Genes Database
GEPIA: Gene Expression Profiling Interactive Analysis.
